# Matrix Rigidity Regulates Cancer Cell Growth and Cellular Phenotype

**DOI:** 10.1371/journal.pone.0012905

**Published:** 2010-09-23

**Authors:** Robert W. Tilghman, Catharine R. Cowan, Justin D. Mih, Yulia Koryakina, Daniel Gioeli, Jill K. Slack-Davis, Brett R. Blackman, Daniel J. Tschumperlin, J. Thomas Parsons

**Affiliations:** 1 Department of Microbiology and Cancer Center, School of Medicine, University of Virginia, Charlottesville, Virginia, United States of America; 2 Molecular and Integrative Physiological Sciences, Harvard School of Public Health, Boston, Massachusetts, United States of America; 3 Department of Biomedical Engineering, School of Medicine, University of Virginia, Charlottesville, Virginia, United States of America; University of Birmingham, United Kingdom

## Abstract

**Background:**

The mechanical properties of the extracellular matrix have an important role in cell growth and differentiation. However, it is unclear as to what extent cancer cells respond to changes in the mechanical properties (rigidity/stiffness) of the microenvironment and how this response varies among cancer cell lines.

**Methodology/Principal Findings:**

In this study we used a recently developed 96-well plate system that arrays extracellular matrix-conjugated polyacrylamide gels that increase in stiffness by at least 50-fold across the plate. This plate was used to determine how changes in the rigidity of the extracellular matrix modulate the biological properties of tumor cells. The cell lines tested fall into one of two categories based on their proliferation on substrates of differing stiffness: “rigidity dependent” (those which show an increase in cell growth as extracellular rigidity is increased), and “rigidity independent” (those which grow equally on both soft and stiff substrates). Cells which grew poorly on soft gels also showed decreased spreading and migration under these conditions. More importantly, seeding the cell lines into the lungs of nude mice revealed that the ability of cells to grow on soft gels *in vitro* correlated with their ability to grow in a soft tissue environment *in vivo*. The lung carcinoma line A549 responded to culture on soft gels by expressing the differentiated epithelial marker E-cadherin and decreasing the expression of the mesenchymal transcription factor Slug.

**Conclusions/Significance:**

These observations suggest that the mechanical properties of the matrix environment play a significant role in regulating the proliferation and the morphological properties of cancer cells. Further, the multiwell format of the soft-plate assay is a useful and effective adjunct to established 3-dimensional cell culture models.

## Introduction

The control of epithelial cell (EC) differentiation and proliferation is critical for tissue homeostasis [1], [2]. EC proliferation is regulated by complex interactions with the surrounding microenvironment, including exposure to growth factors, contact with adjacent cells, and adhesion to components of the extracellular matrix (ECM) [3]–[6]. Alteration of the signaling pathways that regulate the response to these microenvironmental cues is a critical event in tumor initiation, progression and metastasis.

The mechanical properties of the ECM have been identified as an important factor regulating the differentiation and proliferation of a multitude of cell types both *in vitro* and *in vivo*. Specifically, the rigidity (“stiffness”) of the ECM, defined by its elastic modulus (*E*) in units of force per area (Pa), affects the growth, differentiation, and functionality of many cell types, including stem cells, fibroblasts, glial cells, and cardiomyocytes [7]–[11]. In addition, disease states are often accompanied by a local increase in ECM rigidity [12], [13]. Cancer progression in soft tissues is typically associated with an increase in rigidity due to local accumulation of a dense, crosslinked collagen matrix allowing detection of the tumor by physical palpation [14], [15]. Accordingly, nontumorigenic mammary epithelial cells, which normally reside in the soft (*E* = 150 pascals [Pa] or N/m^2^) microenvironment of the breast, show increased proliferation when cultured on stiffer matrices (*E* = 4500 Pa), along with increased migration, augmented ERK signaling, and loss of cellular polarity [16]. These attributes are considered hallmarks of tumor cells and are characterized as being an integral component of a transition from a relatively quiescent to a “malignant” phenotype, driven by a local increase in ECM rigidity [16].

The extent and variability to which human cancer cell lines are responsive to variations in microenvironmental rigidity is unclear. Fibroblasts transformed with oncogenic H-Ras no longer show inhibition of growth on soft substrates [11]. In addition, the growth properties of clonal populations of the breast cancer cell line MDA-MB-231 differ in response to rigidity, and they correlate with the ability to grow in the soft lung or stiff bone *in vivo* [17]. This suggests that the growth properties of a particular cancer cell line in response to substrate rigidity may be determined by its genetic or epigenetic composition.

Analysis of human cancer cell lines is generally performed using cells cultured on rigid plastic, or in Matrigel or soft agar, the mechanical properties of which are poorly defined and/or difficult to modulate. In this study we have adapted a method for culturing cells on biologically relevant “soft” substrates using ECM-conjugated polyacrylamide (PA) gels that can span the stiffness range of 100 Pa–150,000 Pa. We used a recently developed 96-well assay system that arrays PA gels of varying stiffness in user-defined increments across the plate. This system was used to determine how changes in the rigidity of the ECM modulate the biological properties of tumor cells, including growth, morphology, and migratory properties. The cell lines tested diverged into two categories based on their proliferation profiles: “rigidity dependent” lines generally exhibited increasing cell growth as extracellular rigidity increased, while “rigidity independent” lines grew equally well across the entire tested spectrum of matrix stiffness. Importantly, cells which grew poorly on soft gels also showed decreased spreading and migration under these conditions. We assessed the growth of four representative cell lines selected from these two categories *in vivo* by introducing the cells into the soft tissue environment of the lung. The two rigidity-independent cell lines (PC-3 and mPanc96) grew well in soft (lung) tissue, while the rigidity dependent cell lines (A549 and MDA-MB-231) did not grow well in the lung. The lung carcinoma line A549 responded to culture on soft gels by expressing the differentiated epithelial marker E-cadherin and decreasing the expression of the mesenchymal transcription factor Slug. These observations suggest that the mechanical properties of the matrix environment play a significant role in regulating the proliferation and the morphological properties of cancer cells, and that the “rigidity profile” is an intrinsic property of each cancer cell line.

## Results

### Rigidity-dependent growth of cancer cell lines

**Figure 1:**
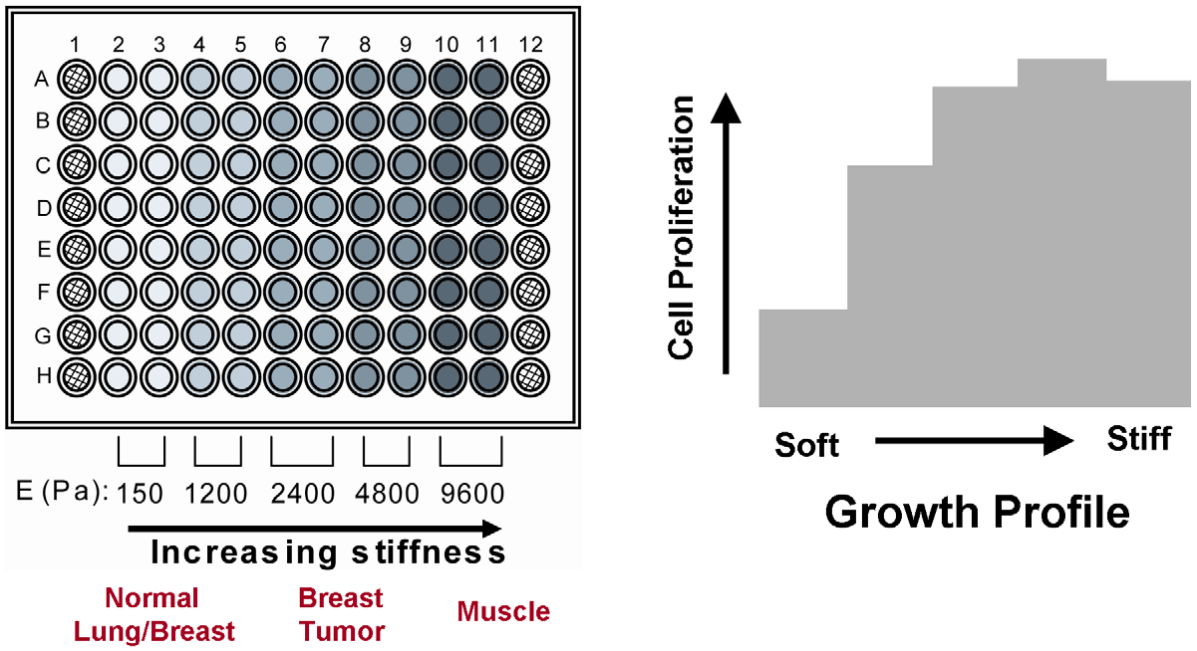
Design of the SoftPlate96 assay. A typical 5-day growth assay using a soft-plate96 yields a “growth profile” which reflects the effect of rigidity on the proliferation of the cell line.

To measure the growth of cancer cell lines as a function of matrix rigidity we adapted a novel 96-well assay system (“soft-plate96”) that uses collagen covalently coupled to polyacrylamide gels as substrates in place of ECM-coated rigid plastic. The soft-plates were comprised of five sections, each containing two columns of collagen-coated PA gels of a specific elastic modulus (Fig. 1), 150 Pa and 1200 Pa (comparable to lung and breast), 2400 Pa and 4800 Pa (comparable to a mammary tumor), and 9600 Pa (approximating striated muscle). These elastic moduli were chosen based on published measurements of the rigidity of soft tissues and tumors [7], [10], [16], [18], and on preliminary data showing that the greatest changes in rigidity-dependent cell proliferation occurred between 150 Pa and 4800 Pa (data not shown).

**Figure 2:**
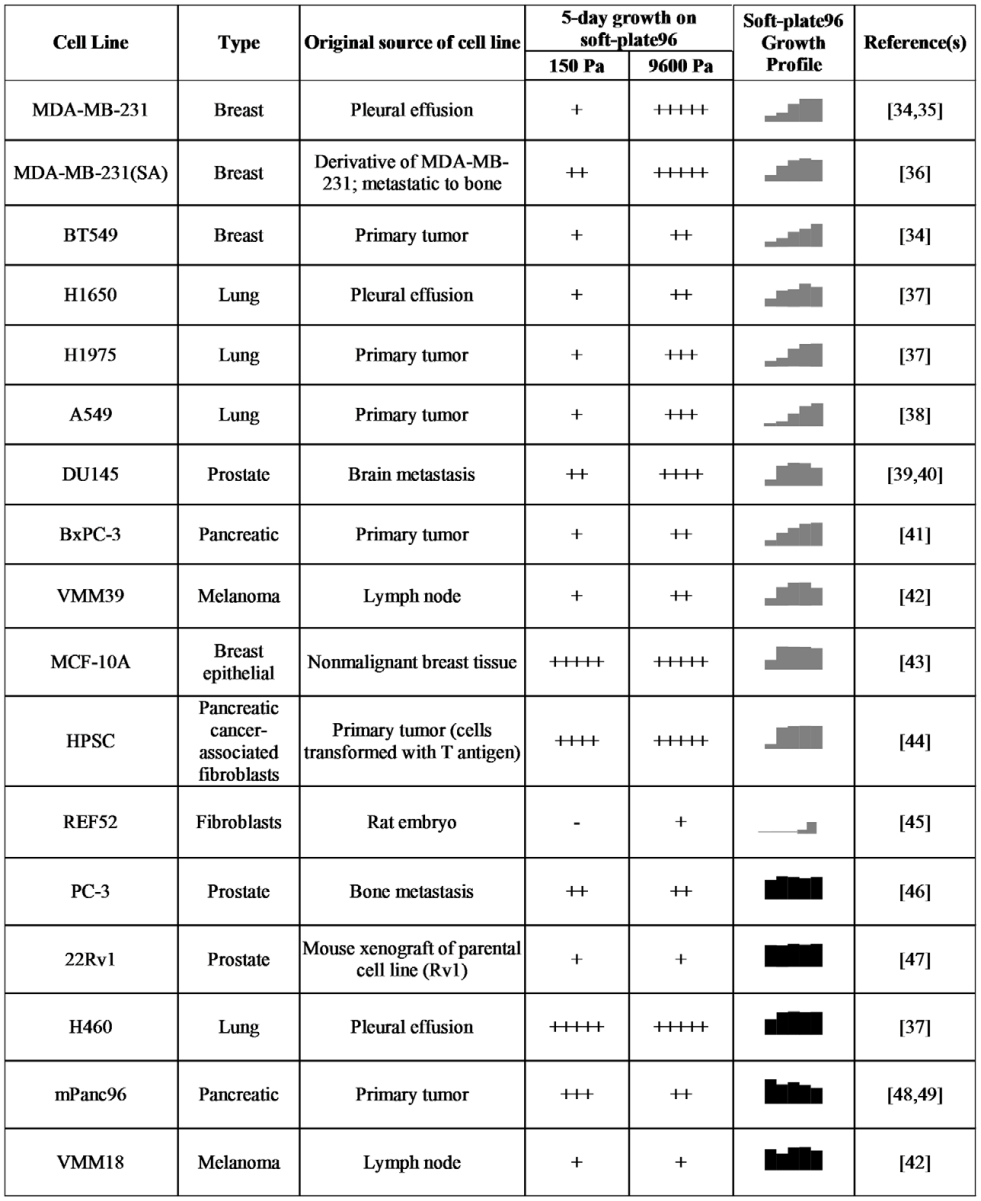
Soft-plate96 growth profiles of cancer cell lines. The table is a compilation of 5-day growth assays for 17 cell lines. Included in the table are original source of the cells (indicated by literature citations), the ability to grow on 150 Pa and 9600 Pa substrates (from SoftPlate96 assays), and the soft-plate96 growth profile for each cell line. Grey profiles indicate rigidity-dependent lines and black profiles indicate rigidity-independent lines. Growth is measured as follows: −<1 fold; +1–5 fold; ++5–10 fold; +++ 10–15 fold; ++++ 15–20 fold; +++++ >20 fold increase in cell number over 5 days.

We determined the growth profile of fourteen cancer cell lines by plating the cells on the soft-plate96 and measuring the fold change in cell number after five days using a fluorescent DNA-binding dye (Fig. 2). In addition, the growth profiles of nontumorigenic mammary epithelial cells (MCF-10A) and two fibroblast lines were determined. Cell growth on defined matrices generated a qualitative “growth profile” for each cell line (Fig. 1, 2). The growth profiles of the cell lines fell into one of two categories: “rigidity-dependent” cells, at least a 2-fold change in cell number across the range of extracellular rigidity tested (e.g., MDA-MB-231 breast cancer cells and A549 lung cancer cells), and “rigidity-independent” cells which grew equally well across the range of tested matrix stiffness (e.g., PC-3 prostate cancer cells and mPanc96 pancreatic cancer cells) (Fig. 2). There was no correlation between the shape of the stiffness-dependent growth profile and the tissue of origin, or whether the cells were originally cultured from the primary tumor or from a metastatic lesion.

**Figure 3:**
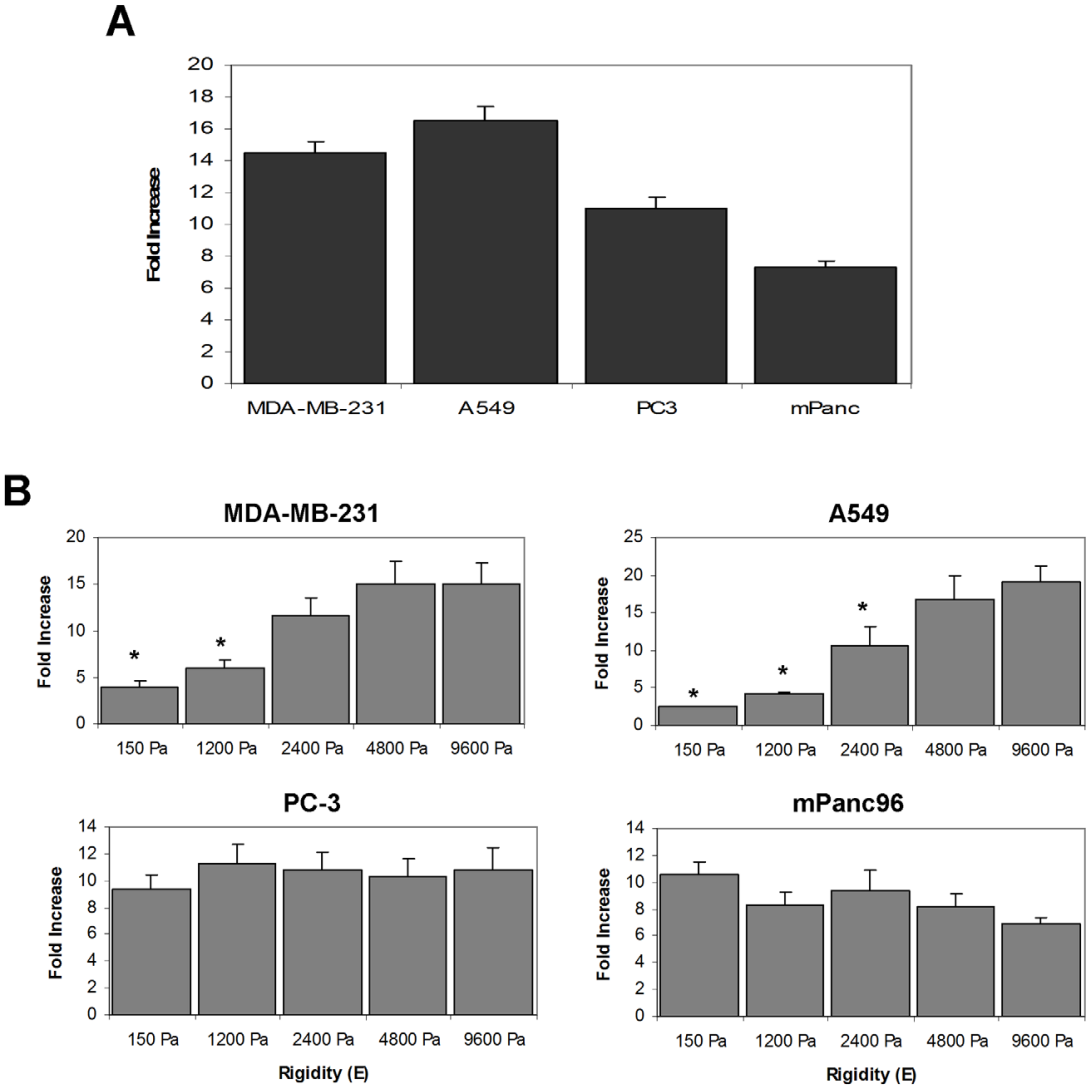
Growth of cancer cell lines on flexible substrates. A.) 5-day growth assay of four cancer cell lines on plastic. B.) 5-day growth assays of the four cancer cell lines on a soft-plate96. Data are expressed as fold change over the number of cells initially plated. Results show mean ± SEM of at least three independent experiments. * p<0.05 vs. growth on 9600 Pa as measured by one-way ANOVA followed by Tukey's test.

We further characterized two cell lines which showed rigidity-dependent growth (MDA-MB-231 and A549) and two cell lines which showed rigidity-independent growth (mPanc96 and PC-3). Each cell line demonstrated robust cell growth on rigid collagen-coated plastic (Fig. 3A). The rigidity-dependent cells demonstrated a 4–5 fold increase in number on the more rigid gels (4800–9600 Pa) relative to the soft (150–1200 Pa) gels (Fig. 3B, top panels). In contrast, the two rigidity-independent cell lines demonstrated nearly equivalent numbers on the soft and rigid gels (Fig. 3B, bottom panels). To determine if the differential growth on soft or rigid substrates represented the selection of a population of cells exhibiting preferential growth on the different substrates, A549 or MDA-MB-231 cells were cultured on plastic or 150 Pa substrates for 15 days. These cells were then harvested and subjected to a 5-day growth assay on a soft-plate96. No change in the soft-plate growth profile was observed after prolonged culturing on soft substrates (Fig. S1). These data clearly establish cell line specific differences in the ability to grow on soft versus rigid substrates and suggest that the “rigidity profile” is an intrinsic property of each cell line.

### Properties of rigidity-dependent and –independent cell lines on different substrates

**Figure 4:**
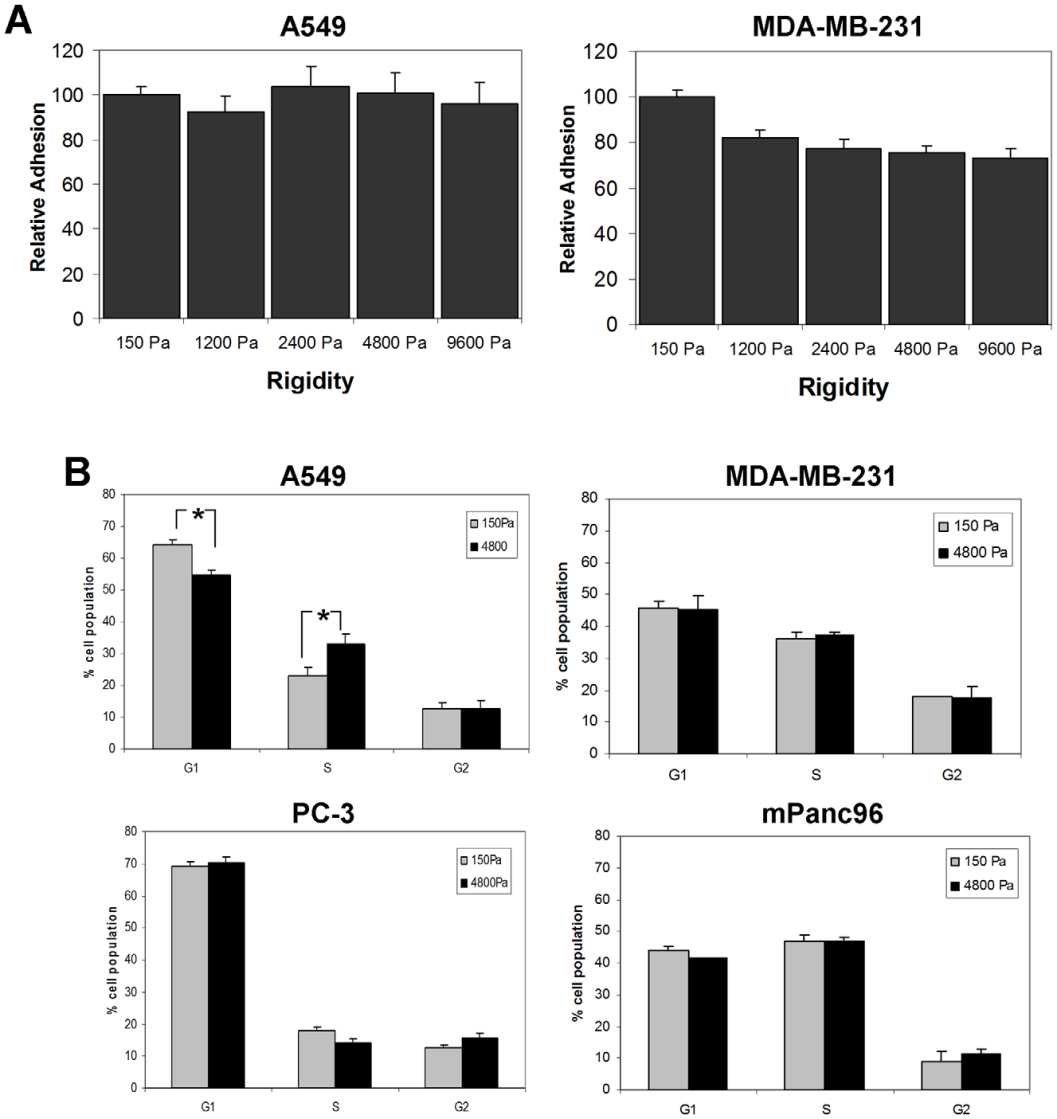
Analysis of adhesion and cell cycle of cancer cell lines on soft gels. A.) A549 and MDA-MB-231 cells were plated on a soft-plate96 and total cell numbers per well were counted after 6 hours of attachment. Data are expressed as percent of adhesion to the 150 Pa gels. B.) A549 and PC-3 cells were cultured on 150 Pa or 4800 Pa gel substrates for 5 days followed by cell cycle analysis. Results show average of at least three experiments ± SEM. * p<0.05.

We assessed whether the decreased growth of rigidity-dependent cells on soft gels was due to defects in adhesion to the substrate, a block in cell cycle, or induction of apoptosis. A549 and MDA-MB-231 cells were plated on the soft-plate96, allowed to adhere for six hours, and the number of attached cells measured. Both cell lines attached efficiently to the collagen-gels irrespective of elastic moduli (Fig. 4A), indicating that lower cell numbers on the gels after five days is not due to a lack of cell attachment.

**Figure 5:**
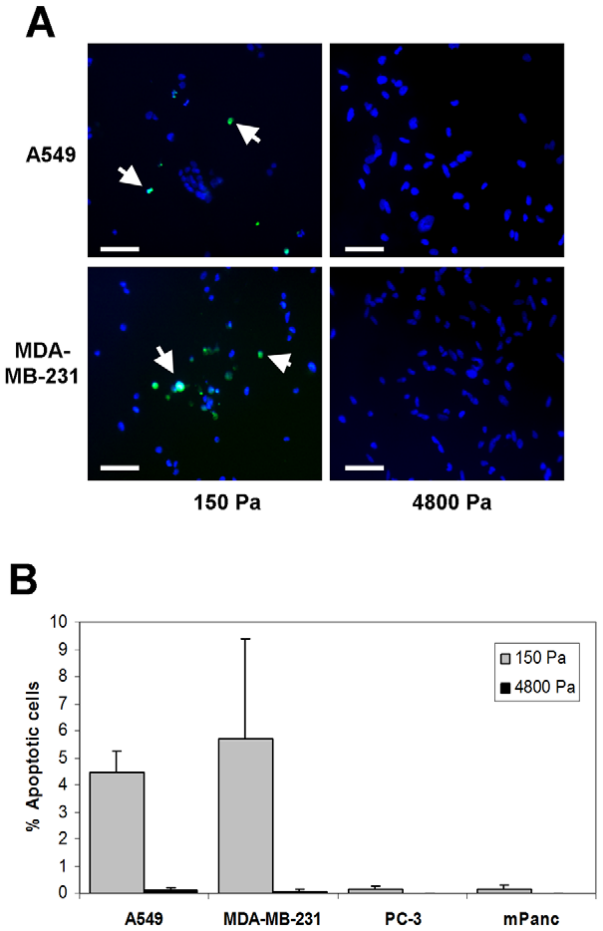
Analysis of apoptosis of cancer cell lines on soft gels. A.) Representative micrographs of A549 and MDA-MB-231 cells plated for 5 days on 150 Pa or 4800 Pa gel substrates. All cell nuclei are stained with DAPI (blue) and TUNEL-positive cells are labeled with fluorescein (green). Bar  = 100 µm. B.) Quantitation of TUNEL staining. Average of 2 experiments ± SEM with a total at least 400 cells counted for each condition.

A lack of adhesion signaling in anchorage-dependent cells results in a block at the G1/S checkpoint of the cell cycle [19]. A549 cells cultured on a 150 Pa gel for five days showed a modest but significant accumulation in the G1 phase of the cell cycle with a corresponding decrease in the percentage of cells in the S phase (Fig. 4B), consistent with a block at the G1/S checkpoint. In contrast, MDA-MB-231 cells exhibited no significant change in their cell cycle profile, similar to the rigidity-independent cell lines PC-3 and mPanc96 (Fig. 4B). However, both of the rigidity-dependent cell lines (A549 and MDA-MB-231) exhibited significant apoptosis when cultured on soft gels for five days, while the rigidity-independent PC-3 and mPanc96 cell lines did not (Fig. 5A–B). None of the four cell lines exhibited significant apoptosis when cultured on the more rigid (4800 Pa) gels. These data indicate that the “rigidity profile” of cells does not reflect differences in adhesion to the matrix, but more likely reflects rigidity-dependent changes in cell cycle progression and cell apoptosis.

### The ability of cancer cell lines to form colonies in soft tissue correlates with their soft-plate96 profiles

**Figure 6:**
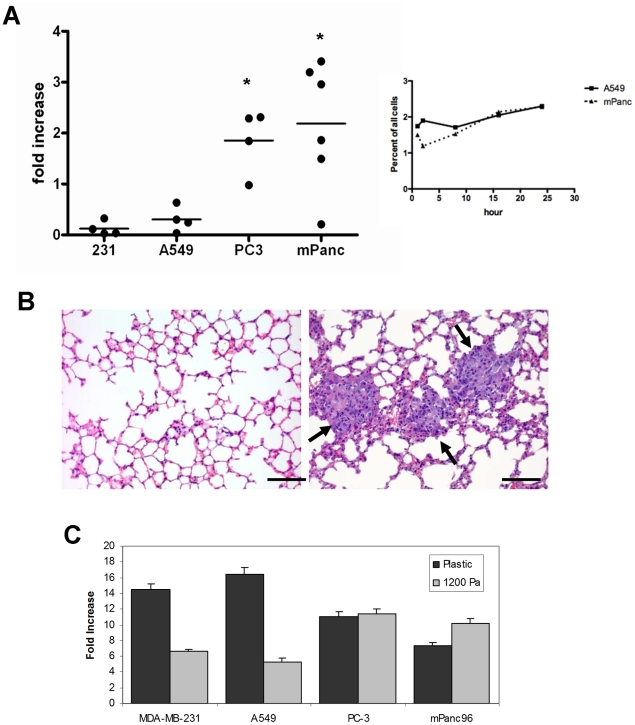
The growth of cancer cell lines in mouse lung tissue. A.) GFP-labeled MDA-MB-231, A549, PC-3, or mPanc96 cells were seeded into the lungs of nude mice. (Left) The number of GFP-positive cells in the lung was determined 4 hours and 14 days after injection, and the change in the number of GFP-positive cells in the lung over the 14 days was scored. * p<0.05 vs. MDA-MB-231 cells as measured by one-way ANOVA followed by Tukey's test. (Right) GFP-labeled A549 and mPanc96 cells were seeded into the lungs and the percentage of GFP-positive cells were scored at intervals over 24 hours. B.) Histology of the mouse lung at 14 days following injection of A549 cells (left panel) and mPanc96 cells (right panel). Arrows indicate micrometastases. C.) Comparison of the growth of cell lines on plastic (taken from Fig. 3A) and on 1200 Pa substrates (taken from Fig. 3B).

We assessed whether the differential ability to grow on soft substrates exhibited by the rigidity-dependent and –independent cell lines was predictive of the ability of these cells to grow in a soft tissue environment *in vivo*. Two rigidity-dependent lines (MDA-MB-231 and A549) and two rigidity-independent lines (PC-3 and mPanc96) were stably transduced with a GFP-encoding lentivirus, and injected into the tail vein of nude mice. Either 2–24 hours or 14 days post-injection the GFP-positive cell population in the lung homogenates was determined by flow cytometry and histochemistry. Each of the cell lines exhibited significant number of cells in the lungs post injection (Fig. 6A, right panel; data not shown). However, after two weeks the lungs of mice injected with the two rigidity-dependent cell lines (MDA-MB-231 and A549) contained fewer GFP-positive cells, compared to the lungs of mice injected with rigidity-independent cell lines (PC-3 and mPanc96) (Fig. 6A, left panel). Hematoxylin and eosin (H&E) staining of paraffin-embedded sections of the lungs two weeks post-injection of mPanc96 cells showed microcolonies within the alveoli, while the lungs of the mice that were injected with the A549 cells and MDA-MB-231 containied no detectable microcolonies (Fig. 6B, data not shown). Thus the growth of the rigidity-independent lines in the lung correlated with their efficiency of growth on the 1200 Pa gels of the SoftPlate96 assay (Fig. 6C), a rigidity similar to that of lung tissue (DJT, unpublished observation). The correlation between relative cell growth rates on soft substrates, but not rigid dishes, with the growth of the same cell lines in the lung suggests that the cells' ability to grow on soft gels *in vitro* may be a predictor of their ability to grow in soft tissue *in vivo*.

### Increased proliferation and cell migration of rigidity-dependent cells correlates with cell spreading

**Figure 7:**
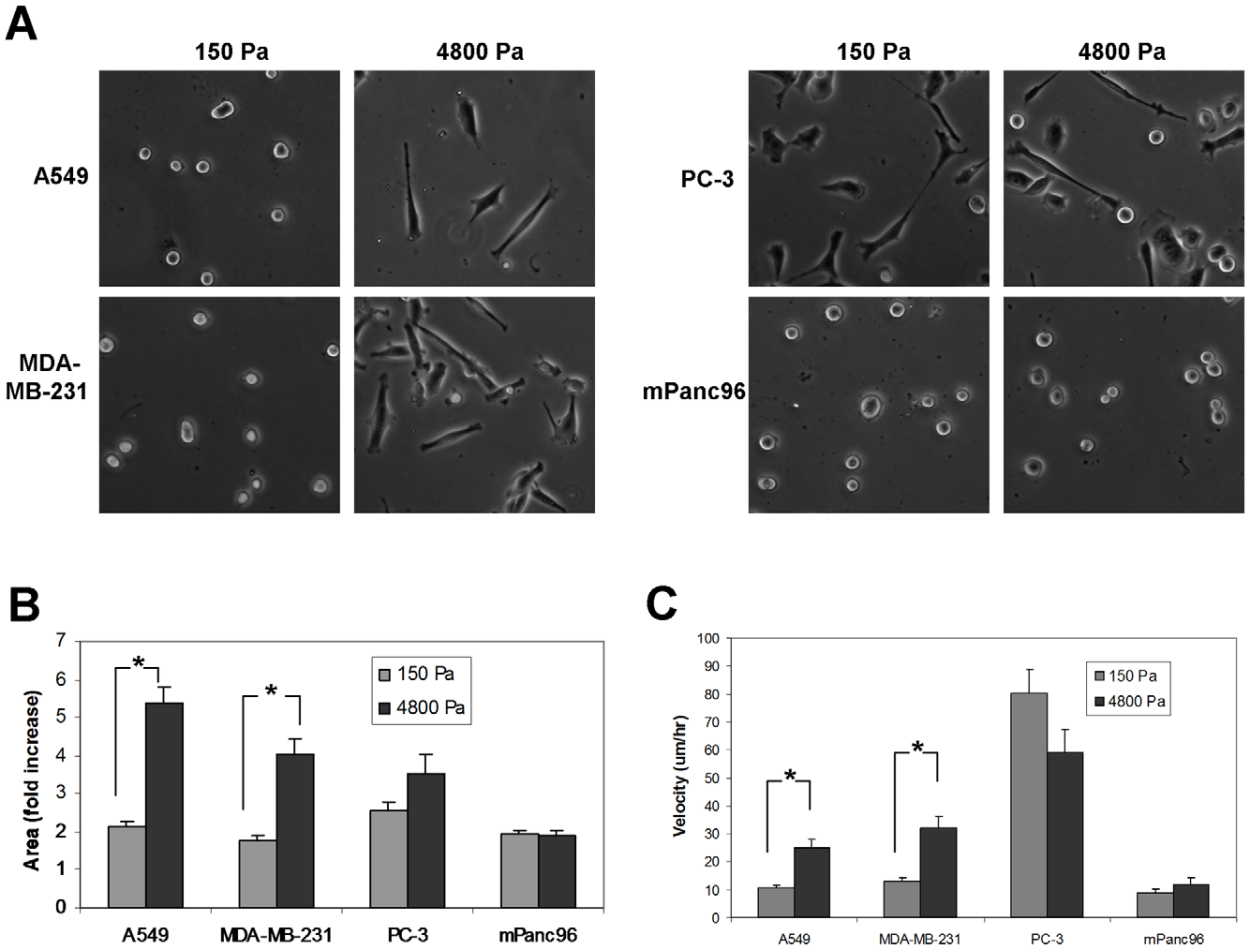
Rigidity-dependent changes in morphology and migration correlate with rigidity-dependent cell proliferation. A.) Micrographs of A549, MDA-MB-231, PC-3, and mPanc96 cells that were plated on 150 or 4800 Pa gel substrates for 20 hours. B.) Areas of cells that were plated for 20 hours on 150 or 4800 Pa gel substrates. Results show mean fold increase over an unspread cell ± SEM of at least 20 cells counted for each condition. C.) A549, MDA-MB-231, PC-3, and mPanc96 cells were plated for 2 hours, then filmed for an additional 18 hours. Mean cell velocity ± SEM in µm/hr was determined by tracing and measuring the paths of 15 cells per rigidity per cell line. * p<0.05.

We next assessed whether proliferation of rigidity-dependent cell lines correlated with the ability of cells to spread on different gel substrates. Both MDA-MB-231 and A549 cells exhibited significant increases in cell spreading on 4800 Pa gels compared to 150 Pa gels (Fig. 7A–B). Similar results were obtained when BxPC-3 cells, a pancreatic line that exhibits a comparable growth profile to MDA-MB-231 and A549 cells (Fig. 2), were cultured on 150 Pa and 4800 Pa gels (data not shown). Interestingly, PC-3 cells (rigidity-independent) were able to spread on 150 Pa gels to a similar extent as on the 4800 Pa gels, whereas mPanc96 cells (rigidity-independent) did not spread appreciably on either soft or rigid substrates (Fig. 7A–B). The ability to spread on more rigid substrates also correlated with the ability of A549 and MDA-MB-231 cells to migrate. In contrast, both PC-3 and mPanc96 cells failed to show significant differences in migration when plated on soft versus more rigid substrates (Fig. 7C). These results demonstrate that for rigidity-dependent cell lines the ability of cells to spread correlates with increased proliferation and migration. For rigidity-independent cells these behaviors appear to be uncoupled.

Focal adhesion kinase (FAK) is a critical signaling component of integrin signaling and has been implicated in sensing the rigidity of the ECM [20], [21]. FAK activity, as measured by its autophosphorylation on tyrosine397, was only modestly activated as a function of matrix stiffness in A549 cells, and was not significantly altered in the other cell lines tested (Fig. S2). These data emphasize that the behaviors of the different cancer cell lines on soft or rigid substrates cannot be simply attributed to alterations in general adhesion signaling through FAK activation.

### The mechanical properties of the microenvironment regulate the epithelial and mesenchymal properties of A549 cells

**Figure 8:**
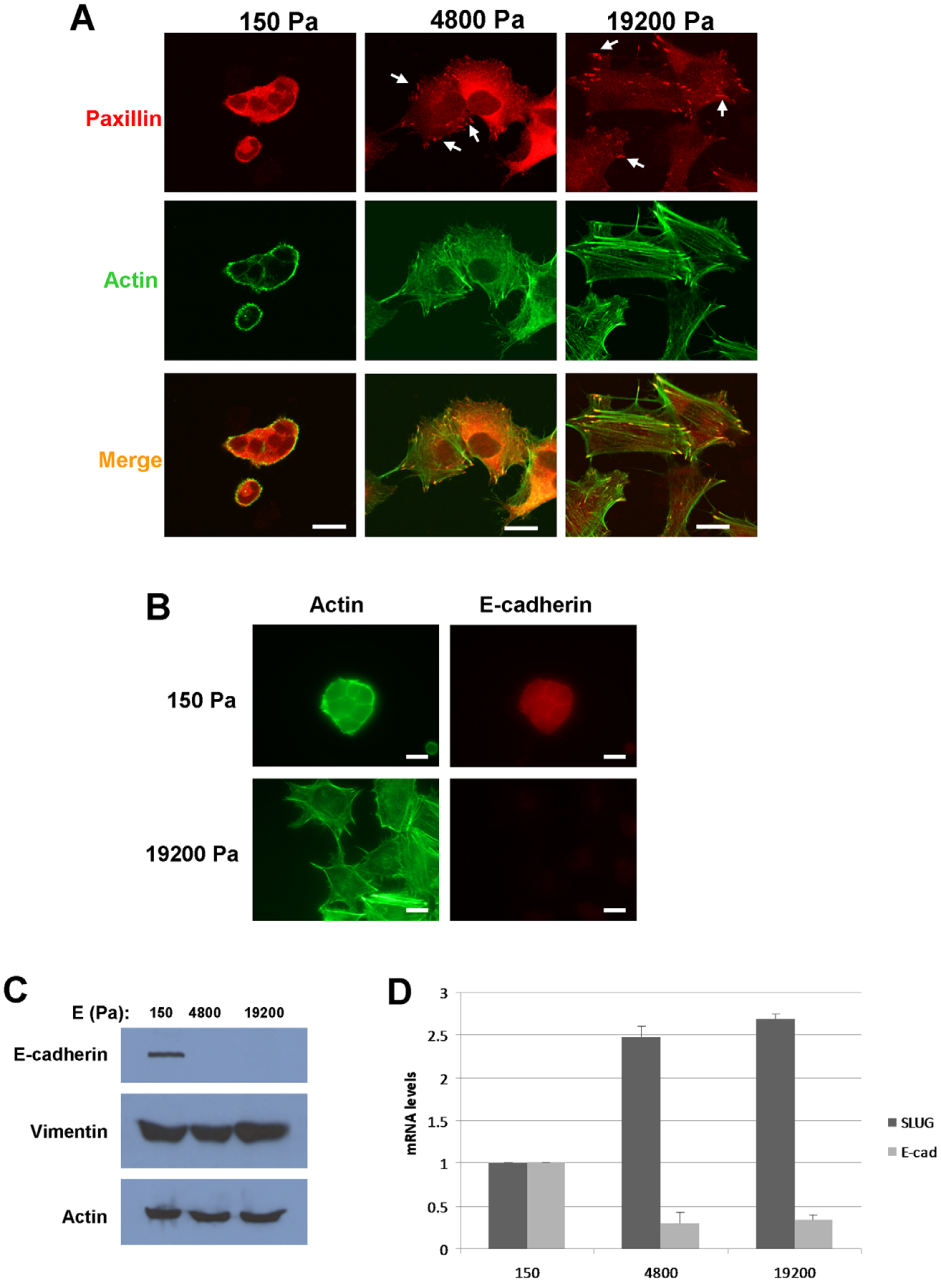
Substrate rigidity regulates E-cadherin expression in A549 cells. A.) A549 cells were cultured for 3 days on gels with rigidities of 150, 4800, or 19200 Pa. Cells were fixed and stained for actin (green) and paxillin (red). Arrows indicate focal adhesions. B.) A549 cells were cultured on gels with rigidities of 150 or 19200 Pa. Cells were fixed and stained for actin (green) and E-cadherin (red). C.) A549 cells cultured on PA gels for 3 days were lysed and blotted for expression of E-cadherin, vimentin, and actin. D.) The relative levels of Slug and E-cadherin mRNA in A549 cells cultured on PA gels for 3 days as measured by real-time RT-PCR. Results show mean ± SEM of three independent experiments.

The conversion of normal epithelial cells to malignant, metastatic counterparts often involves the loss of expression E-cadherin and the acquisition of a more migratory phenotype – a process termed epithelial-to-mesenchymal transition (EMT). A549 cells grown on soft (150 Pa) gels for 5 days formed clusters with no visible focal adhesions or stress fibers (Fig. 8A). In contrast, cells on more rigid (4800 Pa and 19200 Pa) gels were spread and more disperse exhibiting prominent stress fibers and focal adhesions (Fig. 8A), all hallmarks of the mesenchymal phenotype. Immunofluorescence staining or western blot analysis of cells cultured on the 150 Pa gels or 4800 or 19200 Pa gels demonstrated significant upregulation of E-cadherin expression on soft substrates (Fig. 8B–C). However, no significant change in the expression of the mesenchymal marker vimentin was observed in cells growing on the different substrates (Fig. 8C).

The transcription repressor Slug is a member of the Snail family of DNA-binding elements that regulates E-cadherin expression [22] and has been shown to be critical for conferring a metastatic phenotype in an experimental model of melanoma [23]. Quantitative RT-PCR analysis of A549 cells cultured on substrates of different rigidities revealed an upregulation of Slug mRNA when cells were grown on more rigid gels (4800 and 19200 Pa) compared to the soft gel (150 Pa) (Fig. 8D). Slug mRNA levels inversely correlated with E-cadherin mRNA levels, which were lower in A549 cells cultured on the more rigid gels compared to cells cultured on the soft gel, consistent with changes observed in E-cadherin protein expression (Fig. 8B–C). These data indicate that matrix rigidity can modulate E-cadherin and Slug expression in A549 cells. Interestingly, MDA-MB-231 cells, while exhibiting rigidity-dependent proliferation, did not express detectable levels of E-cadherin at either 150 Pa or 19200 Pa (data not shown), suggesting that these cells, while morphologically similar to A549 cells on soft and rigid substrates, do not alter the expression of this epithelial marker when exposed to a soft microenvironment.

## Discussion

The studies outlined above underscore the importance of the mechanical properties of the ECM in regulating cancer cell proliferation and survival. Furthermore, we describe an efficient and flexible assay system to determine how changes in matrix rigidity influence cell properties. Analysis of 14 cancer cell lines revealed that altering the rigidity of the collagen-coated matrix prominently alters the growth of certain cancer cell lines (“rigidity-dependent” growth) while having little effect on other cancer cell lines (“rigidity-independent” growth) which grew robustly even on substrates of very low stiffness. The lower growth rates on soft gels in rigidity-dependent cell lines were caused at least in part by the selective alteration in cell cycle progression and the induction of apoptosis when cell lines were plated on soft matrices. Additionally, the rigidity-dependent lines showed a marked decrease in cell spreading and migration when plated on soft versus rigid substrates, and at least one of the cell lines (A549) exhibited a rigidity-dependent regulation of E-cadherin expression and reversible modulation of epithelial and mesenchymal phenotype. Finally, when seeded into mouse lungs, rigidity-dependent cell lines did not grow as well as rigidity-independent lines, indicating a correlation between the ability to grow on soft matrices *in vitro* and proliferative capacity *in vivo* in the lung.

The soft-plate96 multiwell assay represents a relatively high-throughput approach to assess the role of substrate rigidity on the properties of cancer cells in culture. In this system the method of Pelham and Wang [24] has been adapted to generate a multiwell plate in which the substrate is comprised of polyacrylamide gels of varying stiffness that have been functionalized to provide a binding surface for extracellular matrix proteins, e.g., collagen. In the studies described above the plates were designed to encompass five levels of elastic moduli, ranging from 150 to 9600 Pa, however other formats are easily created. The endpoint of the assay in our studies was cell proliferation, but other endpoints, e.g., cell survival are easily configured. As illustrated, the assay system provides a rapid and reproducible method to assess the role of matrix rigidity on cell growth and survival.

Using this assay we have surveyed a panel of cancer cell lines with the goal of determining how changes in the mechanical properties of the matrix influence cell proliferation. Nine of the cancer cell lines exhibited a dependence on matrix rigidity for growth, growing significantly better on stiff/rigid matrices than on the less rigid/soft matrices. Rigidity-independent cell lines exhibited virtually no changes in growth rate over the range of matrix stiffness used on the plates. Remarkably, all of the cancer cell lines examined in this study were capable of proliferating on soft substrates, whereas normal fibroblasts, smooth muscle and epithelial cells exhibit a strict dependence on matrix rigidity for growth [25]. This presumably reflects the “oncogenic” transformation of the cancer cell lines relative to normal cells, events that reflect the multiple mutations that characterize cancer cells. It is unclear at this time whether the rigidity profile of a cancer cell lines reflects one or more specific mutations that are acquired by an individual cell line.

It is interesting that the cell lines which demonstrated rigidity-dependent growth also showed rigidity-dependent spreading and migration. Our results parallel the analysis of a series of glioma cell lines propagated on fibronectin-coated polymeric substrates of defined mechanical rigidity [26]. On highly rigid substrates (>100 kPa) the glioma cells spread extensively, formed prominent stress fibers and mature focal adhesions, and migrated rapidly. However, when cultured on less rigid matrices (values comparable with normal brain tissue), the glioma cells appeared rounded and failed to productively migrate, similar to the rigidity-dependent cell lines described in our studies. Interestingly, glioma cell motility on highly compliant substrates was rescued by pharmacologic inhibition of actinomyosin–based contractility, suggesting that actinomyosin contractility may be a critical component of the mechanosensory apparatus. Other studies have implicated FAK, ERK, and the small GTPase Rho in the regulation of growth in response to rigidity [16], or an increase in cyclin D levels downstream of Rac activation [25]. Rho GTPases and their downstream targets, which are critical mediators of cell spreading, migration, and contractility [27], may act as mechanosensory machinery that respond to the rigidity of the microenvironment. For example, Rho and its effector Rho-kinase (ROCK) are involved in a feedback loop that regulates tubulogenesis in normal epithelial cells when they are cultured in soft 3-dimensional collagen matrices [28]. When cells are cultured in more rigid, high-density matrices, this feedback loop induces phosphorylation of FAK and ERK, resulting in the increase in expression of genes associated with proliferation, presumably by FAK-dependent Ras activation [20]. While these experiments clearly implicate the Rho pathway in mechanotransduction in normal epithelial cells, further experiments are required to determine the effects of contractility and Rho GTPase activity on cancer cell growth on soft substrates, particularly in cancer cell lines which harbor mutations in the Ras pathway.

As demonstrated in this paper, extracellular rigidity affects the growth of certain cancer cell lines, and the ability of a cell line to grow on a soft substrate *in vitro* may predict its ability to grow in a soft environment *in vivo*. In addition, a cell line's response to extracellular rigidity *in vitro* may predict its reaction to the desmoplastic response *in vivo*, i.e., whether an increase in rigidity of the microenvironment *in vivo* will favor growth of the tumor cells. A cell line's soft-plate growth profile may also predict its sensitivity to therapeutic drugs in soft tissue. For example, the DNA-crosslinker mitomycin C has been shown to inhibit proliferation of mesenchymal stem cells more efficiently on rigid versus soft substrates [29]. In addition, pancreatic cancer cell lines that express epithelial markers such as E-cadherin and lower levels of the mesenchymal marker vimentin are more responsive to erlotinib treatment [30], [31]. Therefore, if a cell line (such as A549) were to become more epithelial-like when cultured in a soft environment, it would be predicted to be more sensitive to erlotinib. Further study both *in vitro* and *in vivo* will be needed to explore the predictive capacity of the soft-plate assay in determining cancer cell responses to *in vivo* soft tissue environments and therapeutic potency within such environments.

Cellular plasticity, or the ability to transition back and forth between a sessile epithelial cell and a migratory mesenchymal cell, is a well-studied phenomenon that is critical to several physiological processes, including embryonic development, wound healing, and cancer progression. A common feature to EMT is a downregulation of cell-cell adhesion, primarily through inhibition of E-cadherin expression, and the acquisition of a motile phenotype along with increased expression of certain infrastructural components such as vimentin. However, this transition may not always be complete, as there are many examples of cells which undergo a “partial” EMT in which cells become motile by transiently acquiring some but not all of the mesenchymal cell characteristics [32]. This suggests that cells undergo EMT in a complex and stepwise manner, and not all EMT events are necessary to achieve a migratory phenotype. E-cadherin expression alone has been linked to inhibition of migration and G1/S cell cycle arrest [3], [6]. Our observations suggest that certain cancer cell lines, while they may have mesenchymal characteristics when they are cultured on rigid substrates, when placed in a soft microenvironment they may respond accordingly by activating an epithelial-type program.

In summary, we have taken a first step in characterizing the response of cancer cell lines to changes in the rigidity of their microenvironment. Further experiments will elucidate the signaling pathways that enable (or inhibit) growth on soft substrates, which may help tailor treatments for tumors based on the mechanical milieu in which they thrive.

## Materials and Methods

### Ethics Statement

The animal studies were carried out in strict accordance with the recommendations in the Guide for the Care and Use of Laboratory Animals of the National Institutes of Health. The protocol was approved by the University of Virginia Animal Care and Use Committee (Protocol Number: 1089). All efforts were made to minimize suffering.

### Cell culture and antibodies

Cancer cell lines were obtained from the ATCC except: MDA-MB-231(SA) cells were a gift from Amy Bouton and Theresa Guise (UVa), VMM18 and VMM39 were a gift from Victor Engelhard (UVa), and HPSC cells were a gift from Rosa Hwang (M.D. Anderson). All cells were routinely cultured in RPMI supplemented with 10% fetal bovine serum (FBS), except for the BT549, 22Rv1, and mPanc96 cell lines, which were maintained in DMEM with 10% FBS. The MCF-10A human mammary cells were maintained as described previously [33]. Monoclonal antibodies to E-cadherin and FAK were purchased from Cell Signaling Technologies. Monoclonal anti-actin and vimentin, and polyclonal anti-FAK pY397 were purchased from Sigma.

### Polyacrylamide substrates

Flexible polyacrylamide substrates were generated on glass coverslips or in 96-well arrays and adapted for cell culture using the method of Pelham and Wang [24]. Polyacrylamide gels contained 3% (150 Pa) or 7% acrylamide (4800 and 19200 Pa), and 0.04% (150 Pa), 0.05% (4800 Pa), or 0.24% (19200 Pa) bisacrylamide. The gels were polymerized on acid-washed, silanated, and glutaraldehyde-treated 22 mm glass coverslips. Each gel was placed in a well of a 6-well dish and activated using the heterobifunctional crosslinker Sulfo-SANPAH followed by coating with collagen I (100 µg/ml) for four hours at room temperature or overnight at 4°C. The gels were soaked in the appropriate growth media at 37°C for at least 20 minutes prior to the addition of cells.

### Soft-plate96 design and fabrication

Glass-bottom 96-well plates (Matrical) were treated with a 0.4% aqueous solution of γ-methacryloxypropyltrimethoxysilane (Acros Organics) to enable covalent attachment of acrylamide to the glass during gel polymerization. Solutions containing 0.075% ammonium persulfate, 0.15% tetramethylethylenediamine, and variable ratios of acrylamide: bisacrylamide (all from Bio-rad) were delivered into the well plate with a multichannel pipettor. A 96-pin block with affixed, hydrophobic glass squares corresponding to the diameter of the wells was inserted, sandwiching the polymerization solutions between two glass surfaces. Gel thickness was controlled by placing 100 µm-thick spacers in the corner wells. Following polymerization, the block was removed and the gels were immersed in 0.5 mg/ml of the heterobifunctional crosslinker sulfosuccinimidyl-6(4′-azido-2′-nitrophenylamino)hexanoate diluted in 50 mM HEPES buffer, pH 8.5. After a 5 minute UV exposure, the crosslinker solution was removed and the gels were rinsed once with HEPES buffer. Monomeric collagen (PureCol) diluted in PBS at 100 µg/ml was delivered to each well and incubated for 4 hours at room temperature. The well plate was rinsed in PBS and UV-sterilized prior to cell seeding.

### Cell growth and adhesion assays

For the cell growth assays, soft-plate96 assay plates were seeded at a density of 1000 cells per well, and the cells were allowed to proliferate for 5 days. The media was changed on the third day. Cell growth was measured on the fifth day using the CyQuant NF cell proliferation assay kit (Invitrogen). Standard curves were generated for each experiment by performing serial dilutions of the cells in an empty row of wells and allowing them to adhere for four to six hours prior to quantitation with CyQuant. For the cell adhesion assay, cells (1×10^3^ per well) in growth media were seeded into a soft-plate96 dish and allowed to adhere for six hours. The media was then removed, the cells were washed once with PBS, and total cell number was determined using the CyQuant assay kit.

### Analysis of cell spreading and migration

For cell spreading analysis, 3×10^5^ cells were plated on gels on coverslips in growth media and allowed to adhere and spread for 20 hours at 37°C. Micrographs were obtained using a Zeiss microscope fitted with a heated stage and a Hamamatsu Orca CCD camera. Quantitation of cell spreading areas was performed using ImageJ by tracing the outline of at least 20 cells in a randomly selected field. To analyze cell migration, time-lapse movies (10 min/frame) were generated of cells (3×10^5^) plated on substrates of different rigidities. Cells were allowed to adhere for 2 hours prior to filming. The nuclei of the cells were tracked over time using the Manual Tracking function of ImageJ, and velocity was calculated by measuring the total distance traveled over time. Cells that underwent mitosis during this period were not traced because they stopped migrating as they divided.

### Cell cycle analysis and apoptosis assay

For cell cycle analysis, cells were cultured on substrates with defined rigidity in growth media for five days. Initial seeding densities were dependent upon the soft-plate96 growth profiles and were as follows: For the A549 cells, 1.5×10^5^ cells were plated on the 150 Pa gels, and 5×10^4^ cells were plated on the 4800 Pa gels. For the PC-3 cells, 7.5×10^4^ cells were plated on each of the 150 Pa and the 4800 Pa gels. The media was changed on the third day of the assay. After five days, the cells were trypsinized or scraped from the surface of the gels and washed twice in ice-cold PBS. The resulting cell pellet was suspended in 70% ethanol and stored at −20°C. Cells were subsequently pelleted and rehydrated in PBS at room temperature for 5 mins prior to incubation in propidium iodide (PI) staining solution (0.1% Triton-X, 20 µg/ml PI, 0.2 mg/ml RNase) at 37°C for 30 mins. Samples were stored at 4°C in the dark until analyzed on a BD FACSCalibur Benchtop Analyzer. Cell cycle analysis was performed with FlowJo v.8.8.6 using the Watson Pragmatic model.

Cell apoptosis was measured using the fluorescent TUNEL assay kit (Roche). Cells were cultured on substrates with defined rigidity for five days, followed by fixation in 4% paraformaldehyde and permeabilization with Triton-X100 in sodium citrate buffer. DNA fragmentation was detected by dUTP end nick labeling as per the manufacturer's instructions. Photographs were taken of random fields and apoptosis (TUNEL positive cells) was scored as a percentage of total cells in each field.

### *In vivo* lung colonization assays

Cancer cell lines were fluorescently labeled by infecting with a lentivirus encoding GFP. 1×10^6^ cells in 200 µl PBS were injected into the tail vein of 6–8 week-old nude mice (Taconic). Lungs were removed at 2–24 hours or 14 days following tumor cell injection and digested in collagenase (0.5 mg/ml in growth media) overnight at 37°C. Lung homogenates were fixed for 20 minutes at room temperature with 2% paraformldehyde. Samples were analyzed by flow cytometry on a FACSCalibur Benchtop Analyzer and data acquired with Cell Quest software (Beckton Dickinson). 5×10^5^ events were collected, and GFP positive cancer cells counted with FlowJo v.8.8.6.

### Cell staining

For immunofluorescence staining, cells were cultured on ECM-coated substrates for three days, washed three times in PBS, and fixed with 4% paraformaldehyde at room temperature for 10 minutes. The cells were permeablized for 2 minutes with 0.5% Triton-X in PBS at room temperature and nonspecific binding was blocked by incubating with 20% BSA and 20% goat serum overnight at 4 C. Immunofluorescence staining was performed at room temperature by incubating the primary antibody with the coverslips for 1.5 hours, washing three times with PBS, then incubating with the fluorescently labeled secondary antibody for 1 hour. The coverslips were washed twice with PBS and twice with water before being mounted on microscope slides and analyzed by fluorescence microscopy. Digital images were captured using a Leica fluorescent microscope equipped with a Hamamatsu Orca CCD camera, or confocal images were captured with a Nikon Eclipse TE2000-E scanning confocal microscope equipped with 488/514 nm Argon and 543 nm HeNe laser lines, using Nikon's EZ-C1 software.

### Preparation of cell lysates for immunoblotting

For immunoblotting experiments, cells were cultured on substrates for three days. The cells were washed three times with ice-cold PBS and lysed by adding sample buffer directly to the gels. The gel was scraped off of the coverslip and heated at 100°C for five minutes. The lysates were cleared from the polyacrylamide by centrifugation through crushed glass wool. Cleared lysates were then separated by SDS-PAGE prior to immunoblotting.

### Quantitative PCR

Total RNA was extracted using the RNeasy Mini kit coupled with RNase-free DNase set according to the manufacture (Qiagen, Valencia, CA). Five hundred nanograms of total RNA was reverse transcribed with the iScript cDNA Synthesis Kit according to the manufacturer (Bio-Rad Laboratories, Inc., Hercules, CA). Quantitative PCR analysis was performed using the CFX96™ Real-Time PCR Detection System and software (Bio-Rad Laboratories, Inc., Hercules, CA). Primers were designed using Beacon Designer software (Premier Biosoft International, Palo Alto, CA). The primers are as follows: Slug forward 5′-CTCCATCTGACACCTCCT- 3′; Slug reverse 5′-ACTGTAGTCTTTCCTCTTCATC-3′; E-cadherin forward 5′ -CCTCTACGGTTTCATAA- 3′; E-cadherin reverse 5′-CTGTATTCAGCGTGACTT- 3′; PSMB6 forward 5′-CAAACTGCACGGCCATGATA-3′; PSMB6 reverse 5′ –GAGGCATTCACTCCAGACTGG-3′. Quantitative PCR was performed with the SYBR Green Supermix (Bio-Rad Laboratories, Inc., Hercules, CA) using 2 µl of cDNA, 300 nmol/L primers in a total volume of 20 µl in triplicates. PCR conditions were 95C for 3 min, followed by 40 cycles consisted of 15 s at 95C, 30 s at 58.7C, and 30 s at 72C for Slug; 95C for 3 min, followed by 40 cycles consisted of 15 s at 95C, 30 s at 53.6C, and 30 s at 72C for E-cadherin; 95C for 3 min, followed by 40 cycles consisted of 15 s at 95C, 30 s at 62C, and 30 s at 72C for PSMB6. Relative expression levels of the target sequences were determined by the standard curve method using cDNA from the A549 cell line which was serially diluted tenfold from 1000 ng to 0.1 ng. Expression levels of Slug and E-cadherin were normalized to PSMB6 (Proteasome subunit beta type-6) as housekeeping gene.

## Supporting Information

Figure S1 Culturing rigidity-dependent cells on soft substrates does not select for a subpopulation of rigidity-independent cells. A549 cells (A, B) or MDA-MB-231 cells (C, D) were cultured on plastic (A, C) or a 150 Pa substrate (B, D) for 15 days. The cells were then subjected to a 5-day growth assay on a soft-plate96. Each cell line exhibited its typical soft-plate profile.
(0.48 MB TIF)

Figure S2 FAK phosphorylation in cancer cell lines cultured on PA gels. A.) Cells were cultured on 150 Pa or 4800 Pa gels for 5 days and FAK autophosphorylation levels were detected by immunoblotting for phospho-Y397 (top) and total FAK (bottom). Numbers refer to fold increase in FAK autophosphorylation over the 150 Pa control. A representative blot is shown. B.) Quantitation of blots as shown in A. Results are a mean ± SEM of at least 4 independent experiments.
(0.70 MB TIF)
