# Supplementary figures and images for: Matrix Rigidity Regulates Cancer Cell Growth and Cellular Phenotype
Source: PLoS One. 2010 Sep 23;5(9):e12905. doi: 10.1371/journal.pone.0012905 (PMC2944843; doi:10.1371/journal.pone.0012905)

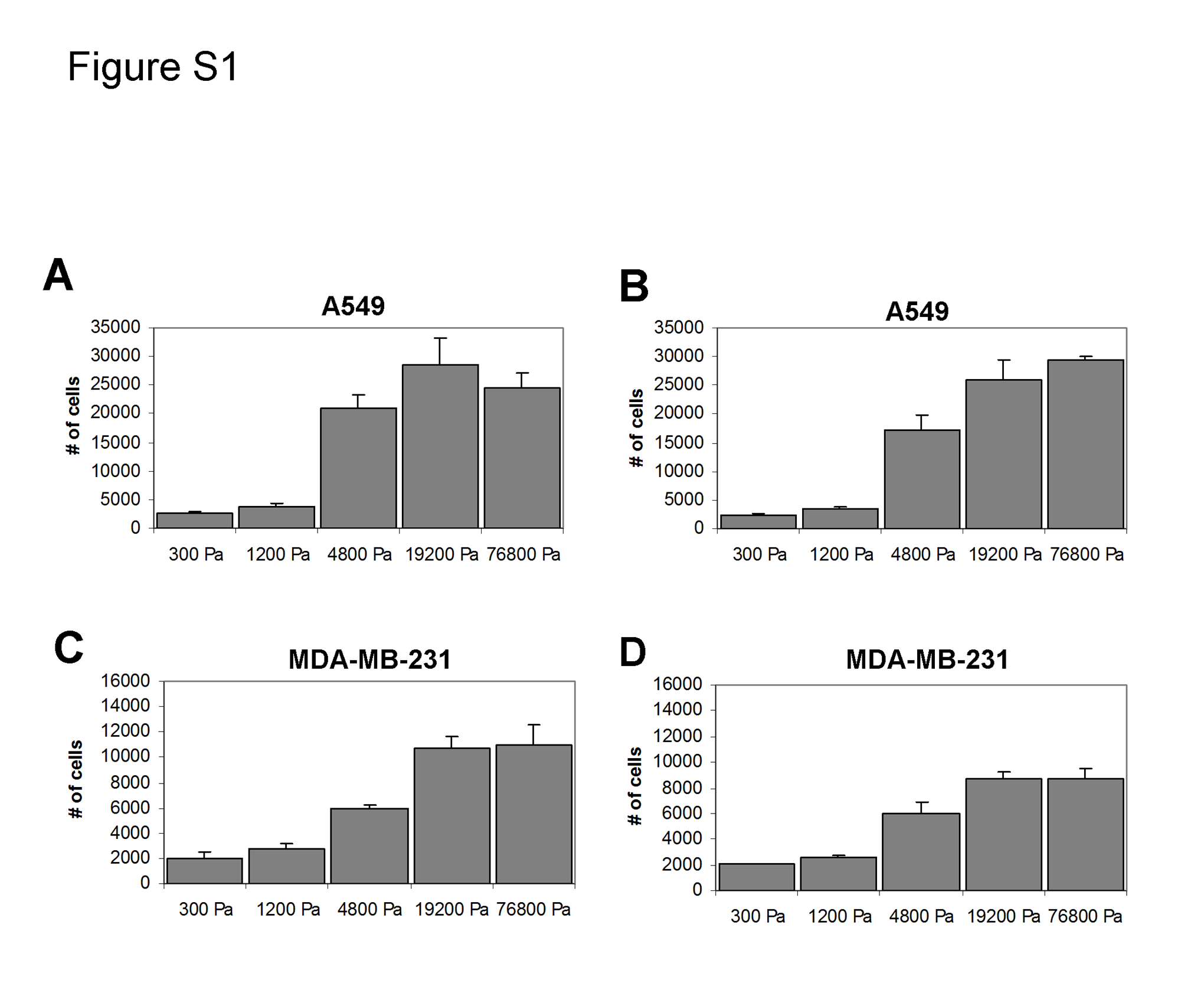

Supplement: Figure S1 — Culturing rigidity-dependent cells on soft substrates does not select for a subpopulation of rigidity-independent cells. A549 cells (A, B) or MDA-MB-231 cells (C, D) were cultured on plastic (A, C) or a 150 Pa substrate (B, D) for 15 days. The cells were then subjected to a 5-day growth assay on a soft-plate96. Each cell line exhibited its typical soft-plate profile. (0.48 MB TIF) [file pone.0012905.s001.tif]

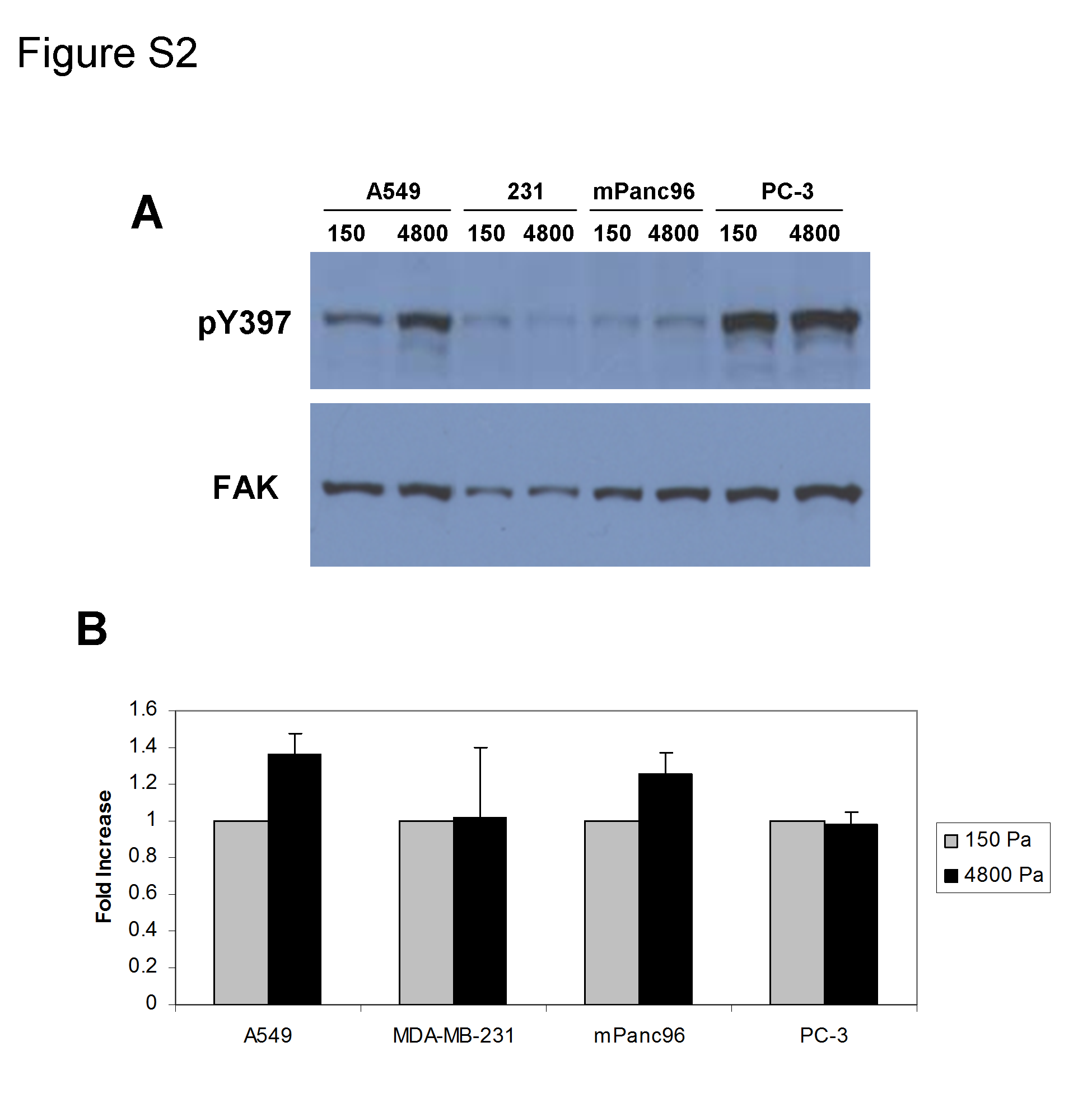

Supplement: Figure S2 — FAK phosphorylation in cancer cell lines cultured on PA gels. A.) Cells were cultured on 150 Pa or 4800 Pa gels for 5 days and FAK autophosphorylation levels were detected by immunoblotting for phospho-Y397 (top) and total FAK (bottom). Numbers refer to fold increase in FAK autophosphorylation over the 150 Pa control. A representative blot is shown. B.) Quantitation of blots as shown in A. Results are a mean ± SEM of at least 4 independent experiments. (0.70 MB TIF) [file pone.0012905.s002.tif]
